# The Value of a Smile!

**Published:** 1916-10

**Authors:** 


### The Value of a Smile!

"Smile awhile, and while you smile another smiles,
And soon there's miles and miles of smiles and life's worth while
because you smiled."

Recently the writer sat in the office of one of the most capable and conscientious physicians in Texas, and as she sat and listened she wondered how the man held his patients—the way he snapped people up over the phone was embarrassing, and it must have given the patients a setback if they had any nerves or self-respect.

A handsomely gowned lady came in for treatment, and when she left the office we drifted out together, and as we sat at lunch a little later our conversation turned to the physician, who had performed a very difficult, dangerous, and unusual operation upon her. "Well," she said, "he is a wonderful surgeon, and one of the most capable and conscientious physicians I have ever known, but he surely is one of the crossest human beings I have ever come in contact with. I go to him for treatment because I know him well, and I jolly him about being cross, but I really wonder he has any patients at all. I surely do feel sorry for his wife." "Has he always been such a 'grouch'?" I asked. "No," she answered, "he is as good as gold at heart, but he has gradually developed into a grouch. He has had ill health and a number of financial reverses, and I think," she added, "he is a very 'poor collector.' "

The day has passed when people feel that a capable man must appear stern and unapproachable. Persons who are in the employ of others are required to "smile and look pleasant," and the public likes it so much that it behooves the person who is working for himself to fall in line, and smile and keep on smiling.

And why should we not smile? There is so much sorrow and suffering and it costs so little to smile and be pleasant and it helps so much.

Recently the writer was given an opportunity to study the magical transforming power of a smile. A small child came into the home; it was decided that all the specialists should examine him, so we started the rounds, eye, ear, nose, throat, stomach, X-ray, oculists, nerve specialists, and so on *ad infinitum*. It was rather trying for a small child, and it soon became evident that the little fellow really dreaded each ordeal, but he just smiled every time he met a doctor, and each time the doctor spoke to him he smiled. Day after day we went the rounds and always the little fellow smiled and smiled, sometimes through tears—but that smile won for him the love and the interest of every physician he came in contact with. Now, what would happen if the physician who has become a "chronic grouch" would smile? Just this, he would have to get a larger reception room, as there would be "standing room only" in the present one, for people much prefer efficiency and cheerfulness to cheerfulness without efficiency, but the decree has gone forth that the man or woman who will win must "smile and be pleasant."

